# Effective elimination of Staphylococcal contamination from hospital surfaces by a bacteriophage–probiotic sanitation strategy: a monocentric study

**DOI:** 10.1111/1751-7915.13415

**Published:** 2019-04-25

**Authors:** Maria D'Accolti, Irene Soffritti, Luca Lanzoni, Matteo Bisi, Antonella Volta, Sante Mazzacane, Elisabetta Caselli

**Affiliations:** ^1^ Section of Microbiology Department of Medical Sciences University of Ferrara Ferrara Italy; ^2^ Department of Architecture and Department of Medical Sciences CIAS University of Ferrara Ferrara Italy

## Abstract

Persistent contamination of hospital surfaces and antimicrobial resistance (AMR) is recognized as major causes of healthcare‐associated infections (HAI). We recently showed that probiotic‐based sanitation (PCHS) can stably decrease surface pathogens and reduce AMR and HAIs. However, PCHS action is slow and non‐specific. By contrast, bacteriophages have been proposed as a decontamination method as they can rapidly attack specific targets, but their routine application has never been tested. Here, we analysed the feasibility and effectiveness of phage addition to PCHS sanitation, aiming to obtain a rapid and stable abatement of specific pathogens in the hospital environment. Staphylococcal contamination in the bathrooms of General Medicine wards was analysed, being those areas the most contaminated and Staphylococci the most prevalent bacteria in such settings. Results showed that a daily phage application by nebulization induced a rapid and significant decrease in *Staphylococcus* spp. load on treated surfaces, up to 97% more than PCHS alone (*P* < 0.001), suggesting that such a system might be considered as a part of prevention and control strategies, to counteract outbreaks of specific pathogens and prevent associated infections.

## Introduction

Persistent contamination of hospital environments by pathogenic microbes is one of the major causes of so‐called healthcare‐associated infections (HAI) (Allegranzi *et al*., 2011), which represent a main concern in all western hospitals. In addition, the frequent antimicrobial resistance of the persisting pathogens, selected by the pressure exerted by the massive use of antimicrobials in hospitals, further threatens the severity of associated infections, which are often rendered untreatable by conventional drugs (ECDC, 2013, 2015). This causes, in the EU alone, about 4 million infections per year, which are directly responsible for about 37 000 deaths each year, and of an economic loss of approximately € 7 billion, including direct costs only (ECDC, 2013).

The control of pathogen contamination has been approached so far by conventional sanitation, based on the use of chemical‐based sanitizers and disinfectants, including chlorine‐derivatives, triclosan, chlorhexidine and others. Although well‐intentioned, this approach has been proven to be unable to decrease microbial contamination in a stable way, as it is not capable of preventing recontamination, which occurs continuously due to the presence of inpatients, medical staff, visiting persons, etc. Other important limitations include the high environmental impact and their potential contribution to the selection of antimicrobial resistant (AMR) strains, thus worsening the AMR concern (Bock *et al*., 2016; Wand *et al*., 2017; Fahimipour *et al*., 2018). Furthermore, chemical sanitizers kill microbes indiscriminately; thus, both the pathogenic and the potentially beneficial normal microbiota are targeted equally. In the search for ideal decontamination systems, we investigated the potential of a probiotic‐based system (PCHS, Probiotic Cleaning Hygiene System), showing that, contrarily to chemical‐based ones, it can decrease in a stable way the pathogen contamination on hospital surfaces (Vandini *et al*., 2014), also leading to reduction in AMR species (Caselli *et al*., 2016) and finally inducing a significant decrease in the risk of acquiring a hospital infection and in the consequent antimicrobial consumption and costs (Caselli *et al*., 2018; Caselli *et al*., 2019). However, because such a system is essentially based on the replacement of pathogens by probiotics through a competitive exclusion mechanism, its action is slow and gradual, and cannot be considered as a rapid mean for the eradication of specifically targeted pathogens, but rather as a preventive and stabilizer system. This type of action might instead be highly desirable when colonized and/or infected patients are hospitalized, or in cases of specific outbreaks, to reduce the risk of infection for subsequent occupiers of the same rooms. In fact, it is known that the presence of such patients increases significantly the risk of contracting that specific infection for patients occupying the same room (Huang *et al*., 2006; Drees *et al*., 2008; Nseir *et al*., 2011), which is due to the persistence of those pathogens in the environment. For this reason, we recently investigated the potential use of lytic bacteriophages, as they are indicated as an interesting safe and green technology for bacterial decontamination, having the characteristic of being extremely specific for individual bacterial strains, and thus potentially usable in a targeted way. Lytic bacteriophages are safe for humans, being able to only infect bacteria and unable to transduce them. In addition, their action is rapid and they can be applied successfully on surfaces, as reported for treatment of food or food‐processing surfaces (Abuladze *et al*., 2008; Tomat *et al*., 2014) and against AMR bacteria (Sulakvelidze, 2005; Jensen *et al*., 2015). Although previously used only against high bacterial densities (Jensen *et al*., 2015), and mostly in aqueous solution (Abuladze *et al*., 2008), our results showed that they can be successfully used to rapidly decrease the amount of pathogens commonly associated with HAIs on different types of surfaces, including AMR strains (D'Accolti *et al*., 2018). Interestingly, the potential to use phages in the hospital environment was recently investigated in intensive care units (ICU), limited to Acinetobacter infections, reporting a significant decrease in Acinetobacter‐associated HAIs when adding a single treatment of ICU rooms with anti‐Acinetobacter phages to the chemical‐based disinfection performed at the patient discharge (Ho *et al*., 2016). Since persistent contamination is a general concern in the hospital environment and not only confined to ICU wards, here we aimed to analyse the feasibility and the effectiveness of a routine phage decontamination in addition to probiotic‐based sanitation.

Towards this aim, based on previous reports by us and others (Marchaim *et al*., 2012; Petlin *et al*., 2014; Caselli *et al*., 2016, 2018), we focused on the most prevalent bacterial contamination in one of the most contaminated areas in hospitals, that is Staphylococcal contamination in ward bathrooms. Importantly, *Staphylococcus* species are directly responsible for the highest percentage of HAIs (Weiner *et al*., 2016), accounting for about 20% of total infections (12% caused by *S. aureus* and 8% by coagulase‐negative staphylococci); infections are particularly severe when sustained by drug‐resistant staphylococci, including methicillin‐resistant *S. aureus* (MRSA), which still represents an important challenge in Europe (Friedrich, 2019). On the other hand, MSRA decolonization of the hospital environment has been reported to have a profound impact on the reduction in MRSA‐associated infections (Edgeworth, 2011). Based on these observations, the sanitation trial was performed in ward bathrooms, having the further advantage of allowing the procedures to be performed without the patients needing to leave the rooms.

## Results

### Study design

**Figure 1:**
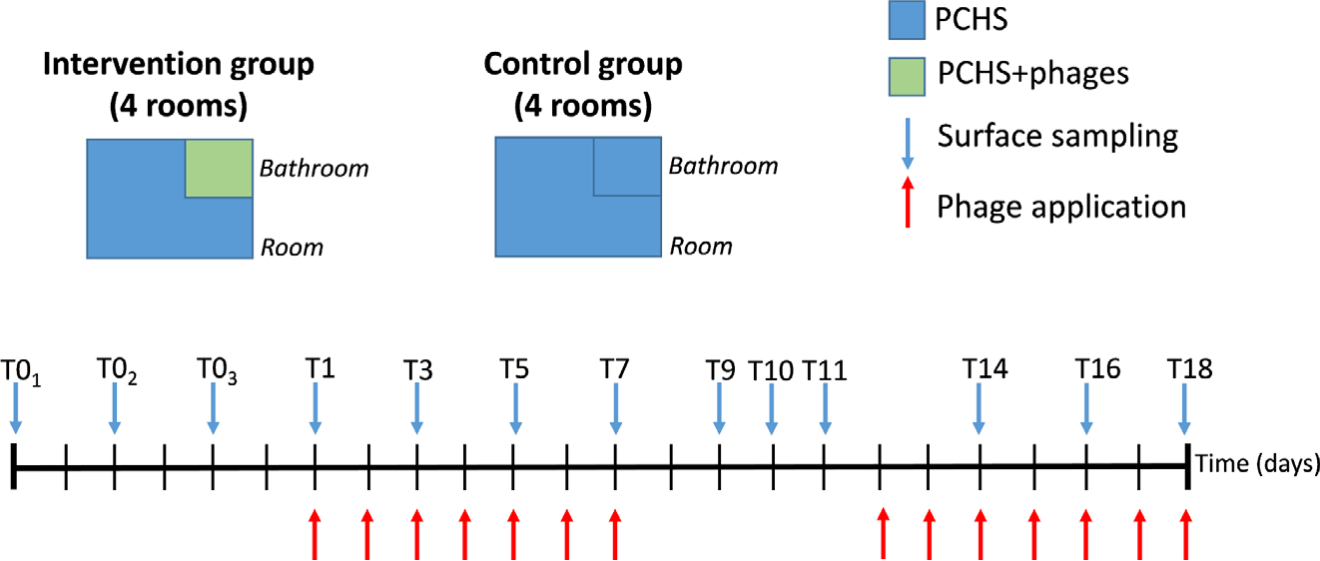
Study design and timeline. Eight rooms of the Internal Medicine ward of the Quisisana Hospital in Ferrara were enrolled in the study and randomly divided into two groups: Intervention group, having the bathroom sanitized by Probiotic Cleaning Hygiene System (PCHS) plus phages; and Control group, having the bathroom sanitized by PCHS alone. Phage applications (red arrows) and samplings (blue arrows) are indicated.

The study was performed in a private hospital located in Ferrara (Italy), after approval by the local Ethics Committee. The trial aimed to analyse the potential effectiveness and feasibility of a sanitation procedure based on the use of bacteriophages directed against Staphylococci, as we previously observed that this type of contamination was prevalent on hospital surfaces (Caselli *et al*., 2016, 2018), with a particularly high level in ward bathrooms. To this purpose, eight rooms of the Internal Medicine ward were enrolled and randomly included in the Intervention group (four rooms) or in the Control group (four rooms). Each room was initially monitored for Staphylococcal contamination for 1 week (three total samplings on alternate days), to quantify the Staphylococcal load. Then, the bathrooms of the Intervention group started receiving sanitation consisting of PCHS plus bacteriophage application, whereas bathrooms of the Control group received PCHS alone. Staphylococcal contamination was monitored for 2 weeks at the following times: days 1, 3, 5, 7, 9, 10, 11, 14, 16 and 18 (Fig.**** 1 **)**. The study design thus included an internal control for each room (comparison between phases in the same room) and parallel controls between groups (comparison between the Intervention and Control groups).

### Characterization of the initial Staphylococcal contamination

**Figure 2:**
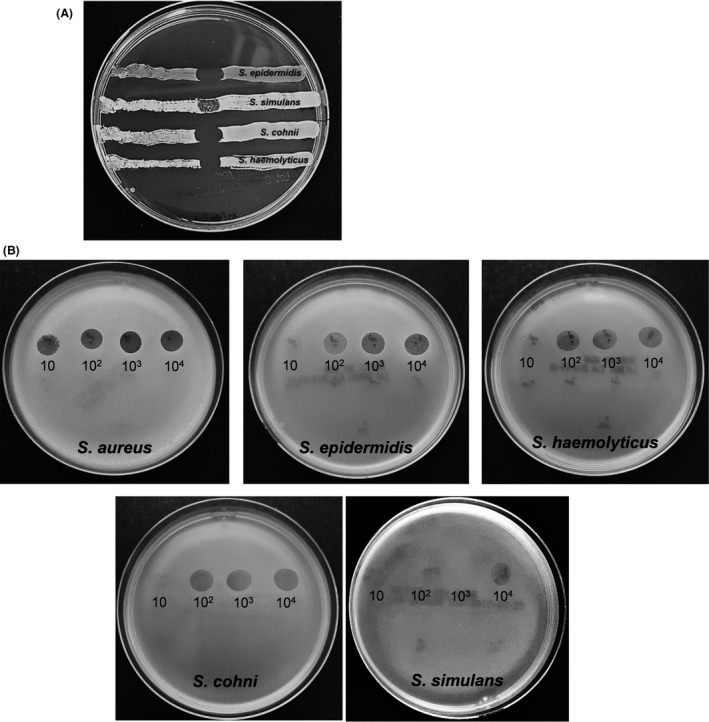
Phage susceptibility test of *Staphylococcus* spp. isolated from hospital surfaces. A. Each coagulase‐negative *Staphylococcus* isolate (in order of abundance *S. epidermidis*,* S. haemolyticus*,* S. cohni*,* S. simulans*) was first analysed for susceptibility to ‘Staphylococcal bacteriophage’ preparation by spot test. B. Both *S. aureus* and non‐*aureus* isolates were tested by soft agar plate assay for their susceptibility to different concentrations of ‘Staphylococcal bacteriophage’ preparation, using 10–10^4^ PFU per spot.

Prior to starting the bathroom treatment, Staphylococcal contamination was quantified and characterized in all enrolled rooms and bathrooms for 1 week on alternate days (three total samplings). Five surface points were sampled by Rodac plates in duplicate: three in the bathroom (bathroom floor, sink and shower plate) and two in the room (room floor and bed footboard). Colony‐forming unit (CFU) counts showed a mean level of contamination corresponding to 3.7 × 10^4^ CFU m^−2^ in the bathroom area (range 0–84 210 CFU m^−2^) and 1.6 × 10^4^ CFU m^−2^ (range 0–41 684 CFU m^−2^) in the room area, respectively, confirming a higher contamination level in the bathroom, as expected. Species identification of the isolated *Staphylococcus* spp., performed by Maldi‐Tof, showed that the majority of isolates were coagulase‐negative Staphylococci, with < 10% represented by *S. aureus*. In particular, we documented four prevalent species, in the order of abundance: *S. epidermidis*,* S. haemolyticus*,* S. cohni* and *S. simulans*. Based on these results, and since the ‘Staphylococcal bacteriophage’ preparation was directed against *S. aureus*, prior to its use on field we tested the susceptibility of each *Staphylococcus* isolate to ‘Staphylococcal bacteriophage’ lysis. Susceptibility tests were performed by spot assays and double‐layer plate assays, including the analyses the most prevalent coagulase‐negative *Staphylococcus* species detected on surfaces, which together with *S. aureus* represented almost the whole Staphylococcal contamination found on tested surfaces. *S. aureus* isolated from surfaces had been already previously characterized for their antibiotic and phage susceptibility, showing that ‘Staphylococcal bacteriophage’ preparation was able to lyse antibiotic susceptible as well as multidrug‐resistant (MDR) isolates in a superimposable way (D'Accolti *et al*., 2018). Both spot and soft agar assay results (Fig.**** 2) indicated that ‘Staphylococcal bacteriophage’ was able to lyse all the *Staphylococcus* species detected on hospital surfaces, although *S. simulans* was affected to a lesser extent, suggesting a broad tropism of such phages towards *Staphylococcus* genus, and allowing their use against the coagulase‐negative staphylococci detected most frequently on surfaces.

### Phage application and impact on Staphylococcal contamination

**Figure 3:**
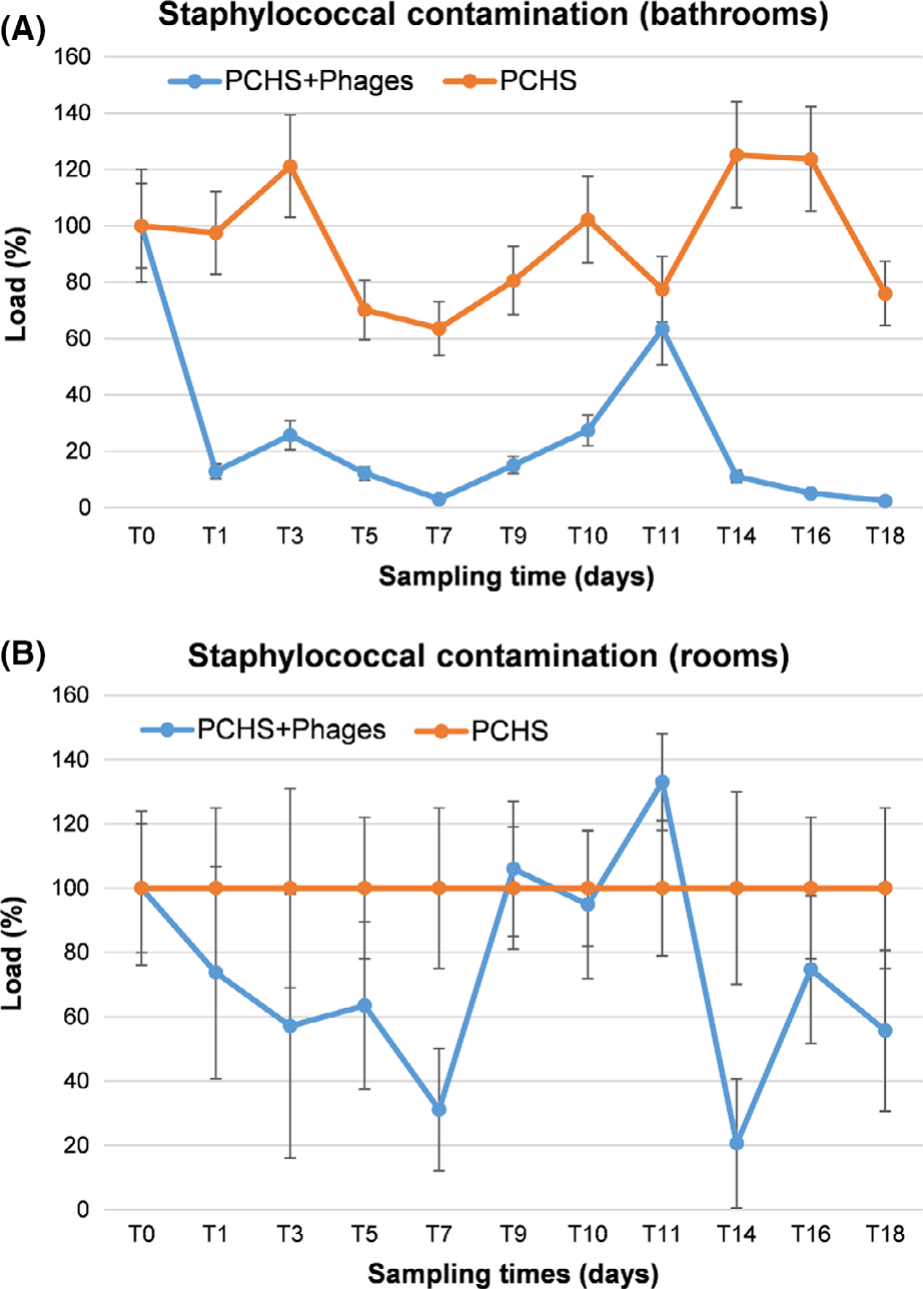
Staphylococcal contamination in enrolled rooms. A. *Staphylococcus* spp. load in enrolled bathrooms. Results are expressed as mean percentage ± SD detected in the Intervention (PCHS + Phages) and Control (PCHS) groups, referred to the initial load, measured at T0, representing 100% value. B. *Staphylococcus* spp. load in enrolled rooms. Results are expressed as mean percentage ± SD detected in the Intervention (PCHS + Phages) compared to Control (PCHS) groups, where each control sample time represents 100% value.

Based on the contamination level measured on hospital surfaces and in previous studies showing optimal phage:target ratio on hard surfaces (D'Accolti *et al*., 2018), a multiplicity of infection (m.o.i.) 1000:1 was chosen for surface treatment, corresponding to ≥ 4 × 10^7^ plaque‐forming unit (PFU) per m^2^ and about 2 × 10^8^ PFU per bathroom (considering a bathroom floor surface of 4.5 m^2^). Surfaces were treated by nebulization. Preliminary tests were performed to optimize the amount of solution needed, to guarantee a homogeneous dispersion on bathroom surfaces and a time of water persistence on surfaces of 10 min, which in previous studies was shown to guarantee optimal contact between phages and bacterial targets (D'Accolti *et al*., 2018). Based on preliminary test results, nebulization was performed for 4 min, using 500 ml of solution. ‘Staphylococcal bacteriophage’ was diluted in filtered PCHS detergent (previously diluted 1:100 in water) (D'Accolti *et al*., 2018), at a final concentration of 4 × 10^8^ PFU l^−1^, and 500 ml (2 × 10^8^ PFU) was used per bathroom. During the nebulization procedure, room inpatients were asked not to use the bathrooms (10 total minutes). Afterwards, bathroom was fully available for patients and staff. Phages were applied daily for 7 days, then discontinued for 4 days and then re‐introduced for a further 7 days. Staphylococcal contamination was assessed by CFU count on Rodac plates at days 1, 3, 5, 7, 9, 10, 11, 14, 16 and 18, monitoring five points in duplicate at each sampling time: bathroom floor, sink, shower plate, room floor and bed footboard. Results showed a rapid decrease in *Staphylococcus* spp. on surfaces following the addition of phages to PCHS sanitation (Fig. 3A). The reduction was already detectable at day 1 (−87%) and was maintained or further increased throughout the following sampling times, until day 7 (−97%). When the phage treatment was discontinued (days 8–11), the *Staphylococcus* spp. load on surfaces gradually increased, although the CFU level remained lower compared to the original load at T0, which may also be due to the action of the probiotics. The re‐introduction of anti‐*Staphylococcus* phages was accompanied by a new, more pronounced, reduction in *Staphylococcus* spp. load on surfaces (days 14, 16 and 18), suggesting that the decrease was actually attributable to phage action. Measured differences were highly statistically significant (*P* < 0.001), except for the T11 sampling time.

**Figure 4:**
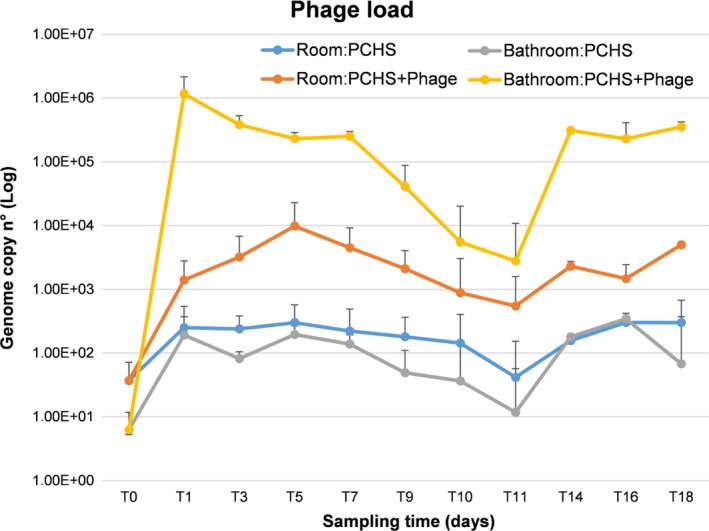
Anti‐*Staphylococcus* phage load in enrolled rooms. Bacteriophage amount on treated surfaces was measured by a specific qPCR performed on collected samples. Results are expressed as mean genome copy number ± SD in rooms and bathrooms of the Intervention (PCHS + Phages) and Control (PCHS) groups.

Interestingly, some reduction in *Staphylococcus* spp. CFU was also observed in the rooms whose bathrooms received the phage treatment in addition to PCHS (Fig. 3B), although the differences between the Intervention and Control groups were not statistically significant. Consistent with contamination data, the CFU decrease in *Staphylococcus* spp. observed on bathroom surfaces was paralleled by the increase in anti‐*Staphylococcus* phages on surfaces, as measured by specific qPCR (Fig. 4). The phage load was in fact significantly higher at all times tested on surfaces of the bathrooms of the Intervention group compared to the mock‐treated bathrooms of the Control group (*P* < 0.001), including days 9, 10 and 11, when the phage treatment was discontinued, suggesting some persistence of residual active phages on surfaces.

Notably, some increase in phages was also detectable in the rooms of the Intervention group compared to Control rooms (*P* < 0.05), suggesting that phages were somehow transported from the bathroom to the adjacent room area. To ascertain whether the decrease in *Staphylococcus* spp. was specific, the microbial population collected from surfaces was further analysed by a qPCR microarray detecting *Staphylococcus aureus, Staphylococcus epidermidis, Enterococcus faecalis, Enterococcus faecium, Escherichia coli, Klebsiella pneumonia/Enterobacter, Acinetobacter baumannii, Proteus mirabilis, Pseudomonas aeruginosa, Clostridium perfrigens, Clostridium difficile, Aspergillus fumigatus, Candida albicans*, total bacteria (*panB*) and total mycetes (*panM*). The results at T0 confirmed the presence of high amounts of *Staphylococcus* spp. (4.1 × 10^4^ ± 2 × 10^3^ genomes m^−2^) and showed the presence of detectable amounts of *E. faecalis* (10^2^ ± 7 genomes m^−2^), *E. faecium* (10^2^ ± 5 genomes m^−2^), *K. pneumonia/Enterobacter* (1.5 × 10^3^ ± 8 × 10^2^ genomes m^−2^) and unidentified mycetes (2 × 10^2^ ± 1 × 10^2^ genomes m^−2^). At T18, analysis results showed a decrease only in *Staphylococcus* spp. genome copies, with no variations in all the other analysed microbes (Table 1).

**Table 1:** Microbial load on bathroom surfaces of the Intervention group

Microbes	T0 (PCHS) (genome copy no)	T18 (PCHS + Phages) (genome copy no)
*S. aureus*	9 × 10^2^ ± 2 × 10^2^	65 ± 54
*S. epidermidis*	4.1 × 10^4^ ± 2 × 10^3^	1.2 × 10^3^ ± 9 × 10^2^
*E. faecalis*	10^2^ ± 7	1.2 × 10^2^ ± 9
*E. faecium*	10^2^ ± 5	0.9 × 10^2^ ± 11
*E. coli*	ND	ND
*K. pneumonia/Enterobacter*	1.5 × 10^3^ ± 8 × 10^2^	1.8 × 10^3^ ± 9 × 10^2^
*A. baumannii*	ND	ND
*P. mirabilis*	ND	ND
*P. aeruginosa*	ND	ND
*C. perfrigens*	ND	ND
*C. difficile*	ND	ND
*A. fumigatus*	ND	ND
*C. albicans*	ND	ND
Total bacteria (*panB*)	7.2 × 10^4^ ± 8 × 10^3^	6.7 × 10^4^ ± 5 × 10^3^
Total mycetes (*panM*)	6.5 × 10^2^ ± 3 × 10^2^	6.0 × 10^2^ ± 4 × 10^2^

ND, not detected.

Results are expressed as mean genome copy number ± SD per m^2^, measured on bathroom surfaces of the Intervention group at T = 0 and T = 18 days.

**Figure 5:**
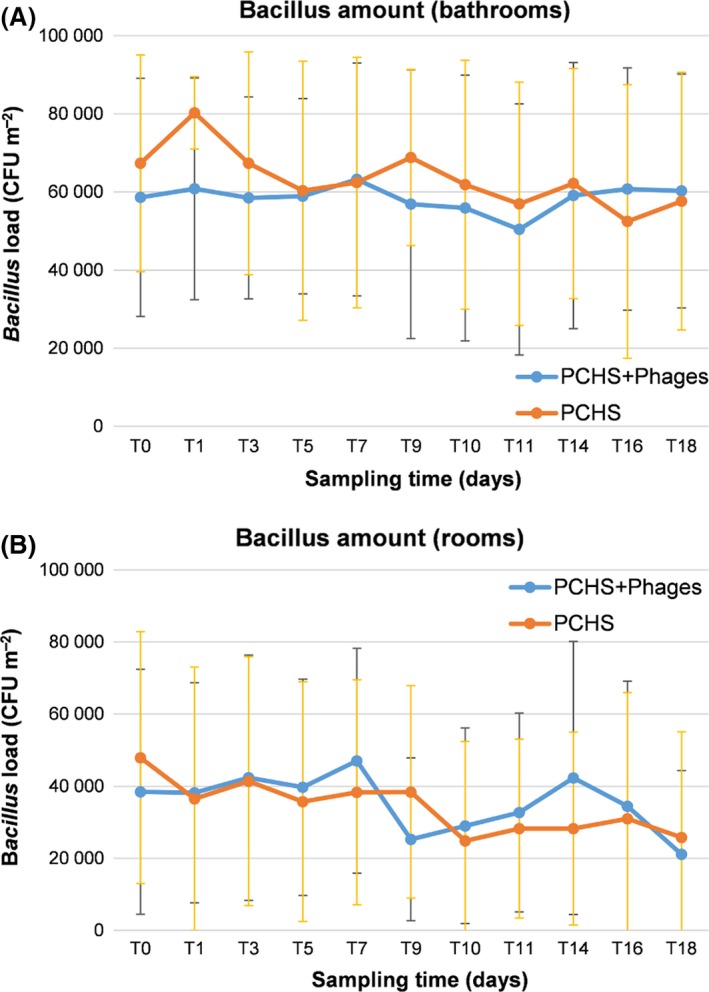
*Bacillus* load in enrolled rooms. The amount of PCHS‐derived Bacilli on sanitized surfaces was assessed by CFU counts on Rodac plates. A. *Bacillus* load in bathrooms. B. *Bacillus* load in rooms. Results are expressed as mean CFU counts ± SD per m^2^, detected in the Intervention (PCHS + Phages) and Control (PCHS) groups.

Finally, we analysed the PCHS‐*Bacillus* load on treated surfaces to assess whether the decrease in *Staphylococcus* spp. number was eventually due to an increase in PCHS‐*Bacillus* amount. PCHS cleanser contained a mix of spores of three *Bacillus* species, namely *B. subtilis*,* B. pumilus* and *B. megaterium*, as already reported (Caselli *et al*., 2016, 2018). The analyses were performed by both conventional CFU count on Rodac plates and by using a specific qPCR amplifying *spo0A Bacillus* gene. The results obtained by direct CFU counts (Fig. 5) showed that PCHS‐*Bacillus* amounts were superimposable on surfaces of the bathrooms of the Intervention and Control groups, as well as in room areas, at all times tested, confirming that the decrease in *Staphylococcus* spp. on surfaces was associated with phage application rather than an increase in probiotic *Bacilli*. Results obtained by qPCR confirmed CFU count results (data not shown).

## Discussion

A stable and specific decontamination action would be highly desirable in a sanitation system, as it would prevent infections associated with the persistence of specific pathogens on hospital surfaces.

The conventional sanitation systems used so far are not able to prevent recontamination, have a high environmental impact and can favour the selection of drug‐resistant strains. By contrast, we recently showed that a probiotic‐based sanitation system (PCHS) can modulate in a stable way the microbiota of treated hospital surfaces, leading to a stable decrease in pathogens (about 80% more than conventional chemical detergents), accompanied by a stable decrease in AMR species (up to 3 log), and to a concomitant halving of the risk of contracting a HAI (Caselli *et al*., 2018).

However, limitations of this system are the long time needed to get a stable microbiota re‐modulation (4–6 weeks) and the lack of specificity against individual targets; both aspects are correlated with the mechanism of action, which resides essentially in the competitive antagonism that Bacilli exert on the growth of other microbes. To overcome these limitations, we hypothesized the use of bacteriophages, based on indications in the literature and on previous results obtained by us (D'Accolti *et al*., 2018). Bacteriophages are in fact characterized by having a very rapid action against specific bacteria and have been proved effective for treatment of food or food‐processing surfaces (Greer, 2005; Abuladze *et al*., 2008; Tomat *et al*., 2014), as well as against various bacterial targets, including *S. aureus* and *E. coli* strains (Sulakvelidze, 2005; Abuladze *et al*., 2008; Jamal *et al*., 2015; Jensen *et al*., 2015; D'Accolti *et al*., 2018). We and others have reported that bacteriophages can effectively attack and lyse their target bacteria independently of their drug‐sensitivity (Sulakvelidze, 2005; Kvachadze *et al*., 2011; Jensen *et al*., 2015; D'Accolti *et al*., 2018), which renders phages a valuable tool to counteract MDR species contaminating hospital environments. Furthermore, we recently observed that phages can be effective against bacterial amounts comparable to those detected on hospital surfaces, that they maintain their activity when diluted in the PCHS detergent already used for hospital sanitation, and that a contact time of 10 min in aqueous solution is sufficient to allow effective contact between phages and target bacteria (D'Accolti *et al*., 2018). Based on these data, we aimed to assess the feasibility and effectiveness of a routine phage application added to PCHS sanitation. To this purpose, we focused on ward bathrooms, as they represent one of the main contaminated areas in the hospital environment, and on *Staphylococcus* spp. contamination, as it represents the most prevalent type of microbial contamination. Eight rooms, randomly subdivided in two groups, were enrolled in the study: the Intervention group had the bathrooms sanitized by PCHS and anti‐*Staphylococcus* phages (‘Staphylococcal bacteriophage’ by Eliava Foundation), whereas the Control group had the bathrooms sanitized by PCHS alone. Staphylococcal contamination of hospital surfaces was quantified and characterized prior to the start of phage application, as phages are specifically targeted towards one or few species belonging to a bacterial genus. The results showed that the majority of contamination was ascribable to coagulase‐negative staphylococci, with *S. aureus* representing < 10% of the total Staphylococci. Therefore, we tested the ability of ‘Staphylococcal bacteriophage’ to recognize and lyse the *Staphylococcus* species prevalently found on surfaces. Interestingly, all the four prevalent *Staphylococcus* species isolated from surfaces (in order of abundance *S. epidermidis*,* S. haemolyticus*,* S. cohni* and *S. simulans*) were efficiently lysed by ‘Staphylococcal bacteriophage’, showing that these phages have a broad and somehow unexpected tropism, as was already suggested in previous reports on Sb‐1 staphylococcal bacteriophage (Kvachadze *et al*., 2011). Phages were applied by nebulization after suspension in filtered PCHS detergent, for the minimum time to assure sufficient contact between phages and target bacteria in aqueous solution (10 min, as determined in previous studies). This also allowed also minimization of discomfort for patients, limiting the time during which the bathroom was not available, and thus allowing a daily treatment.

Surface contamination analyses, following phage introduction, showed an evident additional effect of phages in decontaminating surfaces from target bacteria, with an up to 97% reduction in the Staphylococci number compared to what was obtained using probiotic‐based PCHS sanitation alone. Notably, phage action was very rapid, being immediately detectable at T1 (initial day of the trial) and was maintained for 2 days after discontinuing the treatment, suggesting that it might be effective even when applied on alternate days. As expected, the action was specifically directed towards target bacteria (*Staphylococcus* spp.), and no variations were observed for other contaminating species (Gram‐negative bacteria or mycetes), or in the load of PCHS‐derived Bacilli. Interestingly, some effect was also observed in the rooms having their bathrooms treated with phages, suggesting that phages were somehow also passively transported to contiguous areas, likely by persons walking and/or touching phage‐treated surfaces. Of course, since Staphylococci can also be transported from the bathroom to the room, the diminished bathroom contamination might have had a positive impact on the level of room contamination as well. In conclusion, collected results suggest that a biological sanitation including phage usage might be performed not only for occasional treatment of empty rooms after patient discharge, as previously reported (Ho *et al*., 2016), but also for routine sanitation of specific areas in the hospital wards. This might help to prevent the persistence of high loads of the most frequent surface pathogens, thus diminishing the risk of contracting infections associated with those pathogens. Notably, such a decontamination strategy appears to be economically sustainable, as PCHS costs are comparable with those associated with conventional chemical‐based sanitation, and the additional costs associated with phage production and application impact minimally on PCHS costs. Rather, as PCHS alone was proven to be associated with significant cost savings related to the reduction in HAIs and of consequent antimicrobial therapy (Caselli *et al*., 2018, 2019), the use of a more effective sanitation strategy (probiotic plus phages) might further reduce the infection rate, allowing additional significant cost savings in terms of HAI management.

Notably, phage biocontrol is becoming increasingly accepted as an effective and green technology to prevent and/or eliminate contamination by diverse pathogens in the food field, as well as in water and in agriculture (Jun *et al*., 2016; Moye *et al*., 2018; Svircev *et al*., 2018), in substitution of traditional antimicrobial methods, which kill indiscriminately all bacteria types and have considerable disadvantages, including large initial investment potential damage to surfaces and high environmental impact. Furthermore, the increasing concern of AMR has led to an increasing demand for methods capable of counteracting resistant microbes without worsening AMR emergence, especially in the hospital environment, where such aspects are intrinsically associated with HAI onset and difficult therapeutic approaches.

The recently reported effective use of phages in diminishing the infections associated with the specific phage bacterial target in ICU rooms (Ho *et al*., 2016), together with our results showing that such procedure is usable for daily cleaning purposes, opens the way to future research aimed at evaluation of the impact of a combined probiotic‐phage strategy on specific nosocomial infections.

Although we did not observe a reduction in effectiveness in ‘Staphylococcal bacteriophage’‐ treated bacteria on surfaces, one limitation of our study is the lack of assessment of potential onset of phage resistance in treated Staphylococci. Previous *in vitro* reports showed the appearance of phage resistance with a 1.3 × 10^−8^ frequency for *S. aureus* (Capparelli *et al*., 2007), suggesting that it may be a quite infrequent event. Generally, phage resistance was observed in phage therapy models (Oechslin, 2018), where the density of actively proliferating bacteria is very high. By contrast, bacterial density is much lower on hospital surfaces, where bacterial increase is mainly due to recontamination phenomena (continuous spread of pathogens by inpatients and staff), rather than to proliferation of contaminating species, thus rendering even more unlikely the onset of phage resistance. However, future studies should be addressed to analyse any potential risk to develop resistance to phages in the pathogens contaminating treated surfaces.

In conclusion, being that phages are completely safe for humans, so much so that they are used for therapeutic purposes, we think that such systems might be considered as a part of prevention and control strategies, to prevent frequent infections, or as a tool to counteract outbreaks of specific pathogens in specific settings.

## Experimental procedures

### Setting and ethics statement

The study was performed in the private hospital Quisisana (Ferrara, Italy), after approval by the local Ethics Committee (December 12, 2016) and authorization of the Hospital Medical Director (July 13, 2017). The whole hospital has been routinely sanitized since 2014 by the probiotic‐based PCHS system (Caselli *et al*., 2016). The bathrooms of eight rooms of the Internal Medicine ward were included in the study.

### Study design

The effectiveness of two different sanitation methods was compared by evaluating the level of contamination of the bathrooms of eight rooms located in the Internal Medicine ward. The enrolled rooms were all equipped with an internal bathroom of 4.5 m^2^ and were randomly subdivided into two groups: the bathrooms of four rooms received PCHS sanitation plus phage decontamination (Intervention group), whereas the bathrooms of the remaining four rooms continued to receive sanitation by PCHS alone (Control group). The room surfaces were sanitized by PCHS alone in both groups. Staphylococcal contamination was monitored every 2 days until the end of the trial (23 total days).

### Microbiological analyses

Surface Staphylococcal contamination was assessed by CFU counts on Rodac plates. Five points per room were sampled, each point in duplicate: bathroom floor, bathroom sink, bathroom shower plate, room floor and room bed footboard. Rodac plates of 24 cm^2^ of diameter, containing Baird‐Parker agar medium selective for staphylococci (Merck Millipore, Billerica, MA USA), were used for sampling as previously described (Caselli *et al*., 2016, 2018; D'Accolti *et al*., 2018). After sampling, plates were immediately refrigerated and incubated at 37°C for 48 h within 2 h of sampling. At the end of the incubation time, CFUs were counted and expressed as CFU m^−2^. A total of 400 samples were collected and analysed. The same plates were used also for simultaneous *Bacillus* spp. enumeration, as Bacilli can grow efficiently on Baird‐Parker medium.

### Molecular analyses

The same points collected for microbiological analyses were also sampled by sterile swabs, using a 10 × 10 cm surfaces, as already described (Caselli *et al*., 2016, 2018). Swabs were put in sterile tubes containing 0.4 ml of sterile PBS, immediately refrigerated and frozen at ‐80°C within 2 h. At the moment of analysis, samples were thawed and vortexed (3 × 30 s), to detach microbes from the swab. Total DNA was then extracted from the PBS suspension by a commercial kit (Gene All, Tema Ricerca, Italy), following the manufacturer's instructions.

Three different molecular assays were performed on extracted DNA: total bacterial quantification, PCHS‐*Bacillus* quantification and characterization of microbial contamination and anti‐*Staphylococcus* bacteriophage quantification. Total bacterial load and *Bacillus* quota were evaluated, respectively, by a panbacterial (*panB*) and a *Bacillus*‐specific (*spo0A*) quantitative real‐time PCR (qPCR), as described (Caselli *et al*., 2016, 2018). Characterization of the microbial contamination was performed on bathroom surfaces only, by a customized array assessing simultaneously the presence of the following microbes: *S. aureus, S. epidermidis, E. faecalis, E. faecium, E. coli, K. pneumonia/Enterobacter, A. baumannii, P. mirabilis, P. aeruginosa, C. perfrigens, C. difficile, A, fumigatus* and *C. albicans* (Qiagen, Hilden, Germany). Bacteriophage load on treated surfaces was measured by a specific qPCR designed in the ORF79 (major capsid protein) gene of the Staphylococcus Sb‐1 phage genome (NCBI Ref. Seq. NC_023009.1). Primers, probe and conditions were as follows: forward primer SB‐1(F) 5′‐GTG ATA TCT CAC GCC GTC C‐3′, reverse primer SB‐1(R) 5′‐TTT GGG TCA GAT ACT GGT GC‐3′, probe SB‐1(P) 5′‐FAM‐GTC ATG GTA ACG TAG GTC A‐MGB‐3′; thermal conditions were as follows: 40 cycles, 60°C, using a Quant Studio 5 instrument (Thermo Fisher, Life Technologies). Prior to use, specificity of Sb‐1qPCR was checked by amplifying DNA extracted from bacteriophages targeting different bacterial species, including *E. coli*,* P. aeruginosa* and *S. enteritidis*. No positive reaction was detected with any of the phages other than Sb‐1 phage (not shown).

### Bacterial isolates

Staphylococci population contaminating tested surfaces were characterized by cultural isolation of individual *Staphylococcus* colonies grown on Rodac Baird‐Parker agar plates. Each colony was streaked on a new Baird‐Parker plate and identified by MALDI‐Tof (AccuPRO‐ID; Charles River Lab Europe SaS, Ecully, France). Each isolate was characterized for susceptibility to ‘Staphylococcal bacteriophage’ by spot test and double‐layer plate assays. For spot tests, bacteria in the logarithmic growth phase were seeded on agar plates, and 10 μl of phage preparation was added in a single drop at the centre of the seeded bacteria. Lysis plaques were observed after 24 h of incubation at 37°C. Double‐layer plate assays were performed as previously described (D'Accolti *et al*., 2018).

### Bacteriophages

The concentrated phage product used in this study was obtained from the ‘Eliava Biopreparations’ Ltd. (Tbilisi, Georgia) and consisted of a concentrated solution of the ‘Staphylococcal Bacteriophage’ preparation, that is usually commercially available at the concentration of 10^7^ plaque‐forming units (PFUs) per ml. Instead, the concentrated phage product produced for this study contained 10^10^ PFU per ml. It was maintained at 4°C until use and titred as previously described (D'Accolti *et al*., 2018). The ability of ‘Staphylococcal bacteriophage’ product to recognize and lyse Staphylococci isolated from hospital surfaces was first assessed by spot assays and then by double‐layer plate tests, as previously described, using 10, 10^2^, 10^3^ and 10^4^ PFU per spot (in 10 μl per spot) (D'Accolti *et al*., 2018). For phage application on hospital surfaces, concentrated ‘Staphylococcal bacteriophage’ was diluted in 0.5 μm filtered PCHS detergent at work dilution (1:100 in water) (D'Accolti *et al*., 2018), obtaining a final concentration corresponding to 4 × 10^9^ PFU l^−1^. Such phage preparation is stable for about a week (D'Accolti *et al*., 2018).

### Sanitation procedures

All enrolled rooms received PCHS sanitation both in the room and in the bathroom, performed as already described (Vandini *et al*., 2014; Caselli *et al*., 2016), whereas bathrooms were treated differently depending on the room group: Intervention group bathrooms received PCHS sanitation and phage treatment, whereas the Control group bathrooms received PCHS sanitation alone. All sanitation procedures were performed in the early morning. After optimizing volume and time of application to guarantee homogeneous distribution and persistence of an aqueous film on treated surface for 10 min (which was previously shown to allow an efficient contact between phages and bacterial targets) (D'Accolti *et al*., 2018), a total volume of 500 ml of solution was nebulized in each bathroom by an atomizer. Patients were asked not to use the bathroom for the time of application, whereas the bathroom was immediately available afterwards. Phage application was performed daily for 1 week (days 1–7), then discontinued for 4 days (days 8‐11) and re‐introduced for 7 additional days (days 12–18).

### Statistical analyses

Statistical analyses were performed using parametric Student's *t‐*test and assuming as statistically significant a *P* value at least < 0.05. Analyses were performed using the IBM SPSS 23^®^ software (IBM Corporation, New Orhard Road, Armonk, NY, USA).

## Conflict of interest

None declared.
